# Manufacturing and Preliminary Testing of Nano-Filled Elastomeric Film Cover for Morphing Airfoil

**DOI:** 10.3390/s25165008

**Published:** 2025-08-13

**Authors:** Monica Ciminello, Filomena Piscitelli, Ruggero Volponi, Salvatore Ameduri

**Affiliations:** 1 Adaptive Structures Department, The Italian Aerospace Research Centre (CIRA), 81043 Capua, Italy; s.ameduri@cira.it; 2 Composite Manufacturing Department, The Italian Aerospace Research Centre (CIRA), 81043 Capua, Italy; f.piscitelli@cira.it (F.P.); r.volponi@cira.it (R.V.)

**Keywords:** nanomaterials, elastomer matrix, high deformation, morphing

## Abstract

In this paper, a strain–temperature sensor with medium-high stretchability is proposed for aeronautic applications. The elastomer is conceived to be used as a protective cover on a morphing airfoil characterized by high curvatures. The main novelties in design and manufacturing compared to the state of the art are: use of a non-commercial, low-viscosity PDMS crosslinked with TEOS and DBTDL to enable effective graphene dispersion; innovative sensor design featuring an insulating interlayer on the substrate; and presence of micro-voids to enhance adhesion to the substrate. The resistive performance of the nano-filled matrix is preliminarily verified through a basic functionality test during tensile and bending solicitation at room temperature first and then by considering a thermal cycle while imposing a fixed curvature. During tensile tests, the sensor could withstand an imposed elongation of 30%. The bending tests highlighted the capability of the sensors to withstand low curvature radii, lower than 7.5 cm. Then, within the thermal characterization between −20 and +50 °C, a stability of the signal was observed. A basic resistivity (zero strain) of 3.69 MΩ over a sensor 20 mm long (distance between the electrodes), 5 mm wide, and 1 mm thick. All these features make the sensors a good candidate for laboratory prototypes of morphing concepts. Among the most critical applications in the morphing field, one recalls the possibility of integrating many spots of such sensors at the leading-edge zone of a wing, monitoring the strain at extreme curvature points.

## 1. Introduction

Integrated temperature–strain sensors have attracted attention due to increasing demands in wearable electronics [1,2] in shape reconstruction applications or human motion detection [3] which all require sensing capabilities in such a way that cannot be performed by conventional technology [4,5,6].

Sensors for reconstructing the shape are based on their relative positions. FBG sensors, as a type of embedded sensors, are widely used for the shape-sensing of soft continuum robots [7,8] mainly attributed to their multi-segment sensing characteristics, compact size, anti-interference of electromagnetic signals, and minimal effects on stiffness.

But the principal showstoppers are the difficulty to improve the strength and stretchability simultaneously and the inability to conform to arbitrary surface shapes.

Specifically speaking of the aerospace scenario, some future vehicles and robots would possess adaptive capabilities, from morphing to active deployable properties. That kind of structure is generally characterized by large elongation and twisting. Examples of prodromes in biologically inspired morphing aircraft include, among others, the AFTI/F-111 mission adaptive wing (MAW) with its variable camber wing [9], the SARISTU adaptive trailing edge [10], and NASA’s hyper-elliptic cambered span (HECS) wing [11].

In the cited applications, preliminary attempts have already been made to monitor the morphed shape and the structural health during testing using traditional sensors [11,12] located in a specific region of interest. Furthermore, the traditional metallic and semiconducting sensors show excellent sensitivity but low flexibility due to a low fracture strain value (<5%). On the other hand, fiber optics sensors (distributed or Bragg array) are still quite expensive and unsuitable for monitoring low curvature without being susceptible to performance degradation.

The recent advances in polymeric materials with high strength and stretchability have already been presented for design and testing. The deposition of thin films of conductive materials on elastic polymer substrates is one of the most commonly used methods to develop flexible sensors [13,14]. Elastic polymers such as polyurethane (PU), polydimethylsiloxane (PDMS), and Ecoflex have been widely used in fabricating flexible electronics due to their stretchable and robust mechanical properties [15].

Three key classes of strain sensors have been extensively studied, including piezoresistive, piezoelectric, and piezocapacitive, depending on the physical property affected by the applied load: electrical resistance, charge, and capacitance [16,17].

Among these, piezoresistive sensors are well known for relatively simple readout systems. In the specific case of a polymeric matrix filled with nanoparticles, the conduction of the electrons is due to a complex mechanism that implies their flow in the particle material and at the contact zones among the particles (tunneling effect). When, for example, a stretching is applied, the distance among the conductive particles statistically increases with the consequent diminishing of a certain number of physical contacts, with a net increase of the electrical resistance. In the case of the piezoelectric sensors, the applied strain alters the intrinsic electrical dipoles with a net effect in terms of the electric field. These sensors are not suited for a steady-state regime. Finally, for the piezocapacitive sensors, the applied mechanical load determines a variation of the distance between the opposite conductive films, resulting in a variation of the capacitance.

The previous recap is intended to highlight that most of the research in the field is devoted to the design and manufacturing issues, specifically focused on material optimization. As shown, incredible outcomes are achieved in terms of bulky material elongation (800%) due to the specific rubber matrix [18] that can make it useful for wearable applications. Another piece of information that came from the previous recap is that most of the designed sensors are tested at room temperature (RT) and in quasi-static conditions.

The manuscript at hand reports on a basic research study devoted to the definition of a simple manufacturing procedure for a polydimethylsiloxane micro-structured layer with integrated graphene-filled spots and the preliminary functionality test at RT and short thermal cycle, where the signal is logged and the gauge factor is defined. The main idea is to use the abovementioned layer as a film cover to be bonded to high curvature structures.

The main novelties and cutting-edge aspects in the design and manufacturing of the sensor object of this work concerning the current state of the art are as follows:

1. Utilization of a non-commercial PDMS formulation, specifically a low-viscosity PDMS crosslinked with tetraethyl orthosilicate (TEOS) and Dibutyltin dilaurate (DBTDL), which significantly reduces the overall viscosity of the matrix and thereby facilitates the uniform dispersion of graphene within the polymeric network.
2. Implementation of an innovative sensor architecture, incorporating an insulating interlayer deposited on the substrate before the active sensing layer. This configuration enhances electrical performance and minimizes parasitic interactions between the sensing material and the underlying substrate.
3. Introduction of engineered micro-voids within the sensing layer, which contribute to improved mechanical interlocking and adhesion between the composite film and the substrate, thereby enhancing the overall durability and performance stability of the device.
4. Moreover, such a film can be removed or substituted during maintenance for sensor repair.

## 2. Sensorized Film: Design and Manufacturing

The effective design and manufacturing of a nano-filled elastomer is not a trivial issue. Nanomaterials have a strong tendency to agglomerate, and some dispersion process must be applied. The forces that tend to aggregate the nanostructures become very consistent, causing the sudden increase in viscosity. In addition, due to the electrically insulating nature of the matrix, current does not transit if platelet concentration is lower than a certain threshold (percolation limit). As already mentioned in the previous section, a numerical tool for sensor modeling and conductivity simulation is crucial for driving the manufacturing process. In the paragraph, the main phases, implemented in previous work [19], are briefly described, and the outcomes in terms of resistivity performance prediction are reported.

### 2.1. Performance Simulation

**Figure 1:**
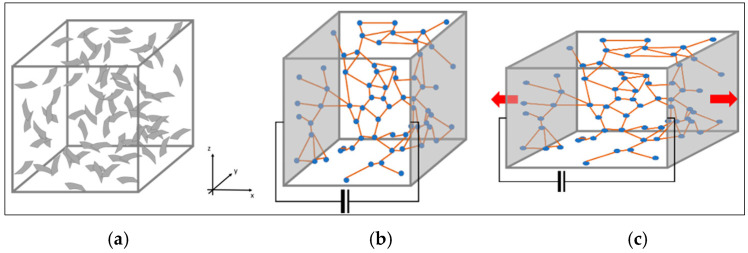
Modeling and simulation phases: random generation of the particle distribution (**a**), equivalent electric network in undeformed (**b**) and deformed (**c**) configurations.

A numerical tool is implemented in Matlab (R2020b) for the simulation of the sensor performance by three operations:

1. Random generation of platelets within a small cubic volume of the matrix:
  This operation is performed by drawing plate geometric elements within a cubic volume. The size of this volume is chosen to include a number of plate elements statistically significant to obtain a repeatability of the electrical performance for different random generations. A cube size 10 times the plate in-plane dimension was considered a good compromise between the repeatability of the performance and the computational effort (strongly dependent on the number of plate elements contained in the volume).
  After the disposition of a random set of plates characterized by certain in-plane dimensions and thickness, an equivalent electrical network was generated, associating at each plate a resistance typical of the graphene material and at each interface zone with close elements a higher resistance, computed through the tunneling effect formula hereafter reported
  (1) $$\rho_{\mathrm{tunneling}}=\frac{h_{p}^{2}}{e^{2}\sqrt{2m_{e}\lambda }}exp\left(\frac{4\pi d}{h_{p}}\sqrt{2m_{e}\lambda }\right)$$
  where
  - *ρ _tunneling_* is the resistivity in the tunneling configuration
  - *e* is the elementary charge (charge of an electron), ≈ 1.602 × 10^−19^ C
  - *m_e_* is the mass of the electron, ≈ 9.11 × 10^−31^ kg
  - *h_p_* is Planck’s constant, ≈ 6.626 × 10^−34^ J·s
  - *l* is the energy barrier height relative to the electron’s energy
  - *d* is the thickness or width of the tunneling barrier
  This relation was used to implement the numerical predictive model of the resistivity of an elastomeric matrix filled with graphene platelets.
2. Change of the original dislocation of the platelets on the basis of the applied strain field and generation of a FE model of the platelet conductive network:
  This operation was addressed assuming the material of the matrix was incompressible and, thus, its volume. In this sense, any stretching along a direction was compensated by a contraction in the other two directions. In practice, said ε_x_ the stretching along the x direction, the corresponding deformation along the other two directions, ε_y_ and ε_z_ were assumed halved and of opposite sign. In line with this assumption, the position of each plate element was changed, thereby obtaining an electrical network corresponding to the deformed case. The resistance parameters were again calculated in this new configuration.
3. Estimate of the voltage drop, normal to the applied strain direction (x), between the faces of the cube and the electrical resistivity/conductivity:
  The estimate of the abovementioned parameters was carried out through the MSC/Nastran software (2024). Since no electric network solution is present in this software, a similitude was implemented. To apply a certain voltage, the temperature boundary conditions were assigned, while the electrical resistance of the elements of the network was simulated by computing an equivalent thermal conductivity. Finally, a thermal analysis was performed to get the unknown heat flow passing through the two opposite faces, representing in this similitude the searched electrical current.
  For the sake of clarity, hereafter, the equation sets solved are reported, both for the thermal analysis and the electrical problem [19].
  (2) $$\bar{q}=\overset{̿}{k_{T}}\cdot \bar{\Delta T}\;\text{thermal analysis basic equation}$$
  (3) $$\bar{I}=\overset{̿}{k_{R}}\cdot \bar{\Delta V}\;\text{electric equation}$$
  where
  - $\bar{q}$ is the heat flux vector corresponding to the current intensity vector, $\bar{I}$
  - $\bar{\Delta T}$ is the thermal gradient vector, corresponding to the voltage potential vector, $\bar{\Delta V}$
  - $\overset{̿}{k_{T}}$ is the thermal conduction matrix obtained by assembling all the finite element of the connections among the platelets; this matrix corresponds to the electrical one, $\overset{̿}{k_{R}}$
  The ratio between the applied voltage and the estimated current represents the resistance of the cubic element filled with conductive nanoparticles.
  The abovementioned operation was addressed both for the undeformed and deformed cases to appreciate the variation of electrical conductivity due to the applied strain. In Figure 1, the three main phases of the simulation are sketched: the generation of a random distribution of platelets with a cubic volume (a), the building of an equivalent electric network with a voltage applied on two opposite faces (b), and the stretching of the volume (c).

**Figure 2:**
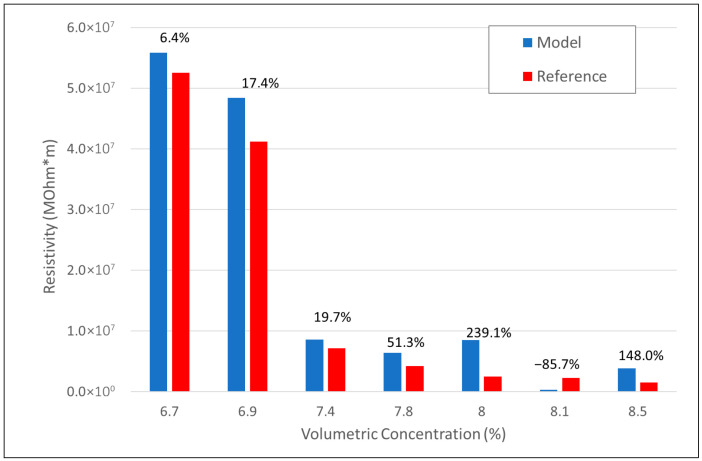
Percolation region: model (red bars) vs. reference (blue bars).

The model handles rectangular platelets and takes into account three main parameters: the length of one side of the platelets, the aspect ratio, and curvature. The model is preliminarily validated with reference literature, in terms of the percolation threshold. To this scope, 100 nm-sided square platelets are modeled. The resistivity vs. vol % predictions are traced and compared with the reference one, achieving a good agreement, as shown in Figure 2. In this plot, the percentage deviation concerning the reference (Equation (1)) was reported on top of the bars.

The major deviations from the reference occur for higher volumetric concentrations (greater than 7.8%). At low concentrations, the transport occurs via tunneling. This phenomenon is captured by the model through Formula (1). However, as the concentration increases, the percolation effect becomes more and more relevant with the formation of physical conductive networks (direct contact between the platelets). In this condition, in fact, the particles tend to aggregate or align, forming clusters or more complex structures such as graphite-like stacks. This means that the platelets tend to be no longer randomly distributed.

**Figure 3:**
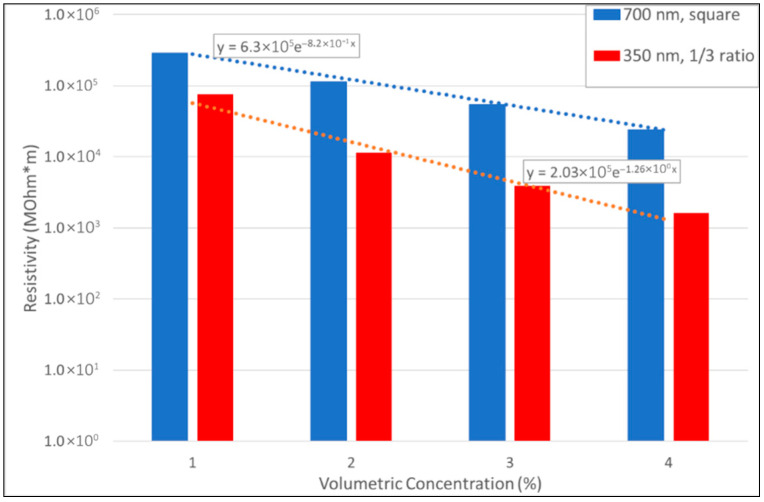
Resistivity/strain % ratio vs. platelet volumetric concentration.

The effect of the introduced parameters is investigated through a parameterization performed on a total of 100 randomly generated platelet-filled volumes. The most promising configuration (here intended as platelet size, aspect ratio, and curvature, assuring the lowest resistivity, for fixed volumetric concentration) is characterized by a flat platelet with an edge of 350 nm and an in-plane aspect ratio of 1/3. The resistivity of this size of platelets is compared in Figure 3 to the particles used for the experimental tests (squares of side 700 nm). An exponential trend line is estimated, and the resistivity at a concentration of 10%, used for the specimens presented in the next section, is predicted. The obtained value is 182 MΩm, corresponding to a resistance of about 3.64 MΩ for a sensor length of 20 mm (electrode distance).

Having verified the possibility of implementing the individuated numbers in the actual manufacturing process (dispersion of platelets), the outcomes are used to ensure the optimal value for the sensor conductivity.

### 2.2. Film Manufacturing

The sensor chemical composition and material characterization can be found in detail in [20], while herein the main phases concerning improvements in the manufacturing process and surface treatment are reported.

The polymeric matrix is prepared by mixing two parts of polydimethylsiloxane (PDMS), OH-terminated, having molecular weights of 110,000 and 550, respectively, Tetraethyl orthosilicate (TEOS), and Dibutyltin dilaurate (DBTDL) at room temperature. The low initial viscosity of the polymeric matrix is achieved by employing PDMS at low molecular weights. TEOS used as a crosslinker contributes to further lowering the viscosity, and then avoids the use of a solvent for the filler’s dispersion. No sonication was addressed, nor were solvents added to improve the dispersion of graphene and further abate the percolation threshold; indeed, it would also have increased the resistivity value of the nano-loaded polymer.

The nanocomposites are prepared by dispersing the graphene powder (10 ÷ 15 wt%) into the polymeric matrix for 30 min by using magnetic stirring. Although the results shown in Figure 2 prove that the resistivity does not change significantly beyond a value of 7.4% of the conductive material for sensors subject to high deformation, it is advisable to have systems loaded well above the percolation threshold, as high elongation can break the conductive paths, rendering the sensor inoperable. This is because the percolation paths would be less dense, meaning that high operating voltages would be required to obtain a detectable signal.

Since the elastomer is conceived to be used as a protective cover on a morphing airfoil, some surface treatment must be considered. Elastomers, by their chemical nature, are low-surface-energy materials. For these reasons, obtaining good anchorage to flexible substrates can present a challenge to release liner producers.

Silicones anchor to substrates by way of two mechanisms: mechanical interlocking with a substrate and chemical reactions with a substrate. Both mechanisms contribute to the anchorage of silicones.

Mechanical interlocking occurs when elastomers are applied to semi-porous substrates such as a rough micro-void surface. Silicon flows very readily and can penetrate easily into porous surfaces, creating a mechanical “bond”.

**Figure 4:**
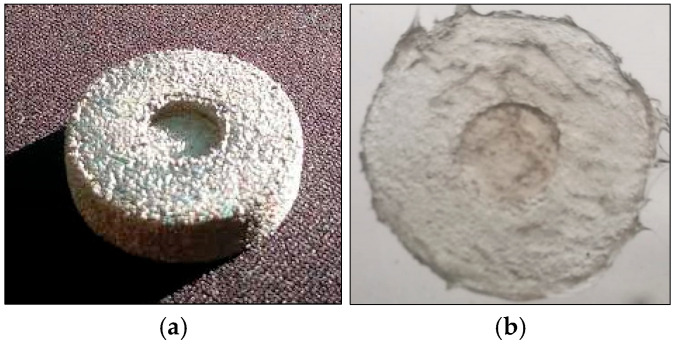
Example of 3D printed mold: (**a**) circular titanium-made mold, (**b**) example of elastomer surface effect.

To provide a good, strong adhesion onto a structural component, a special mold is realized using 3D printing. Titanium powder was used as the raw material, with particle sizes ranging from 45 to 105 μm, and 30 mm × 30 mm × 3 mm blocks prepared by laser additive manufacturing technology. The density of the sample was approximately 4.5 g/cm^3^. Selective laser melted titanium was characterized by low porosity, relatively large surface roughness, and pronounced surface texture (Figure 4a). Elastomer surface shows average roughness (R_a_) not higher than 1.5 μm (Figure 4b).

**Figure 5:**
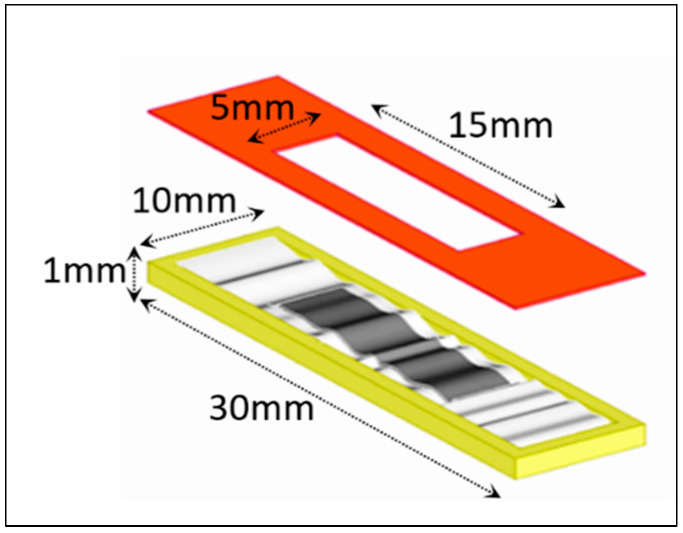
Schematic of the deposition tool: titanium mold (in yellow), ABS mask (in red).

The final tool has been selected with a rectangular geometry, and consists of a titanium mold and an ABS mask used to shape the conductive PDMS-G dispersion (see Figure 5).

To connect the electrodes with the device, in order to minimize interference in mechanical measurements, very thin and flexible copper wires are used. The electrodes are made with conductive paste. The electric resistance (0.43 Ω/m) is negligible with respect to the resistance of the sensor to be measured (order of MOhm).

**Figure 6:**
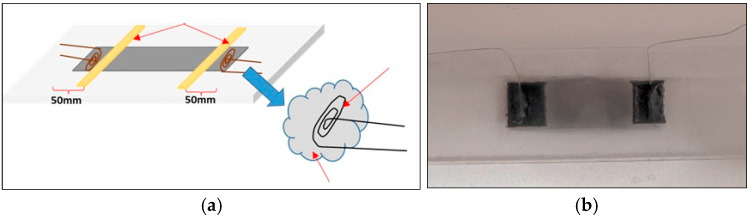
Example of sensor item: (**a**) sketch of electrodes integration by conductive adhesive paste, (**b**) manufactured sensor.

Each electrode is connected by two enameled copper wires. To remove the enamel at the wire ends, the ends themselves are immersed in a molten tin-lead alloy, at a temperature of about 380 °C, for a few seconds, that is, until some floating enamel slag is noticed at the molten alloy surface. Then, one end of the two wires is secured with adhesive tape at each end of the sensor body shown in Figure 6.

## 3. Preliminary Functionality Test

The sensor is mainly tested in two basic load conditions: tensile and bending. The goal of these two basic experiments is the estimation of:

1. max elongation under tensile by reaching the ultimate load.
2. matrix anchoring strength by detecting linear deviations of the signal during bending.

All the experiments have been realized by setting up a hydraulic press control Instron5900R machine, including a load cell up to 100 kN; a climatic chamber Instron3119-607; a high-resolution multimeter Fluke 8508A with an accuracy of ±5 ppm for the resistance provided by the graphene sensor. Then, a PC-interfaced readout unit with HBM QuantumX 1516B for electrical strain gauge and MOI-sm130 for fiber Bragg grating, is used to log raw data from other very common strain sensors used as a reference.

### 3.1. Elongation Performance

**Figure 7:**
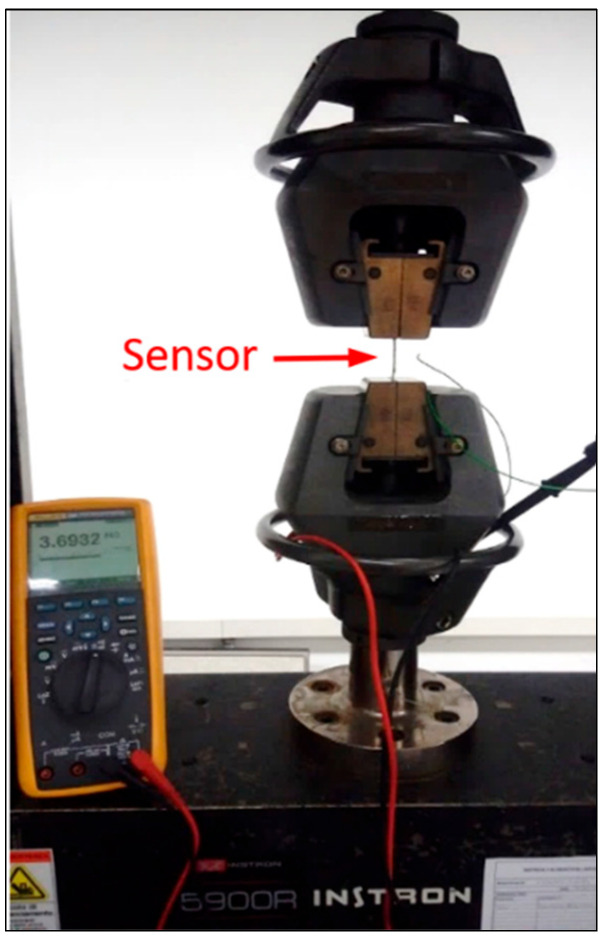
Bulk sensor elongation test.

The sensor is clamped on both sides; the tensile load is applied (Figure 7). The test outcomes are a linear response value up to 30% of absolute elongation. Resistance variation shows a very linear trend with no evidence of local deviations.

**Figure 8:**
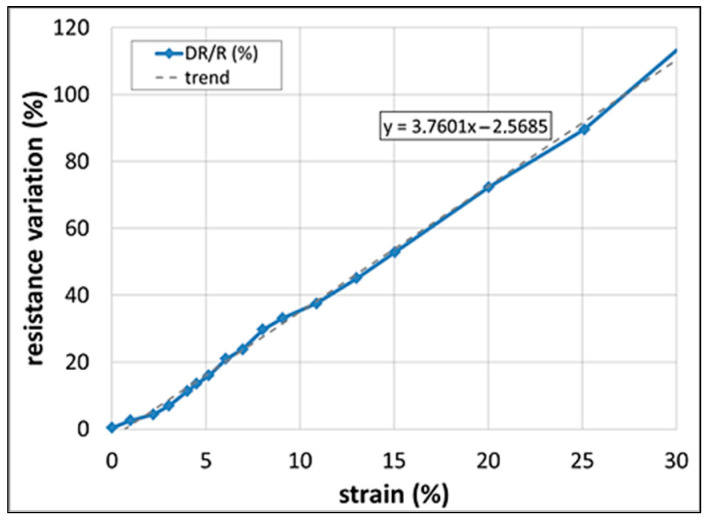
Numerical–experimental comparison of resistive trend during elongation.

The numerically predicted resistance of 3.62 MΩ at zero strain through the trend blue line of Figure 3, is in good agreement with the measured resistance of 3.69 MΩ (Figure 8).

As evident in Figure 8, the resistance increases by about 110% when a maximum strain of 30% is applied, practically passing from an initial value of 3.69 MOhm to a final one of 7.75 MOhm.

### 3.2. Matrix Anchoring Performance

The second test is realized with a sensor foil attached to a metallic surface to estimate how the bonding layer performs in terms of strength adhesion and in terms of gauge factor losses.

A good secondary bonding has been conducted at first by using a degreasing agent on both the elastomer lower surface and the metallic substrate with trichloroethylene solvent.

**Figure 9:**
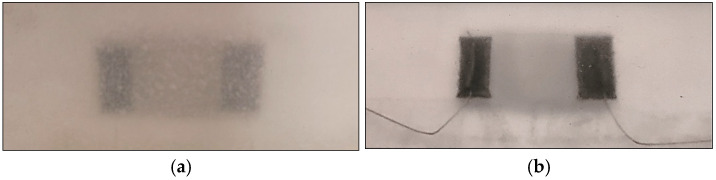
Sensor bonding visual inspection: (**a**) M-Bond 200 adhesive produces an opaque film layer at the interface, (**b**) Master Bond MasterSil 153 adhesive produces a cleaner interface.

Then two kinds of adhesive are applied in a very thin layer: M-Bond200 acrylate common adhesive (Figure 9a) and Master Bond MasterSil 153 (Figure 9b), which is a two-component, paste silicone compound for high-performance bonding.

The visual inspection revealed that M-Bond 200 adhesive produced a brittle and opaque film layer at the interface, while Master Bond MasterSil 153 adhesive produced a cleaner surface.

MasterSil 153 has been selected for the test. It has a convenient one-to-one mix ratio by weight and will not outgas while curing. It is 100% solids and contains no solvents. First and foremost, MasterSil 153, as is true with silicones in general, offers remarkable flexibility and high temperature resistance. It has superb electrical insulation properties. Its remarkable flexibility allows it to withstand severe thermal cycling and resist vibration and shock. The service temperature range for this system is −65 °F to +400 °F.

For the test execution, two thin beams (1.2 mm thick) made of aeronautic aluminum alloy AL7075 and harmonic steel are used. The strain values provided by the reference strain gauge are used for comparison. The sensor has been bonded with the beam in a flat position. Once the beam has been installed in the cantilever position, the resistance value changed to 3.42 MOhm due to the proper mass corresponding to a tip displacement of almost 0.5 cm. This configuration has been considered to offset the strain at zero.

**Figure 10:**
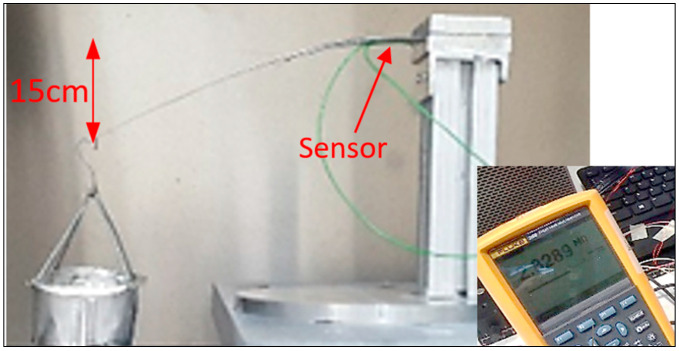
Sensor patch on aluminum beam: resistance value of 2.82MOhm at max tip displacement.

**Figure 11:**
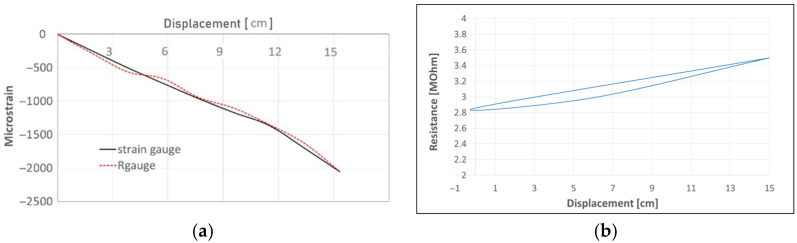
Bonded sensor patch under bending load: (**a**) strain values as a function of the tip displacement, (**b**) resistance variance as a function of the tip displacement.

Then, a max weight applied is 110 g, which is able to produce a tip displacement of 15 cm (Figure 10) and a resistance variation of 2.82 MOhm. The sensor patch and the strain gauge have been collocated on the lower side of the beam to measure compression (Figure 11a). The gauge factor is used to estimate the resistance variance from the initial unloaded position under gravity up to the maximum displacement (Figure 11b).

**Figure 12:**
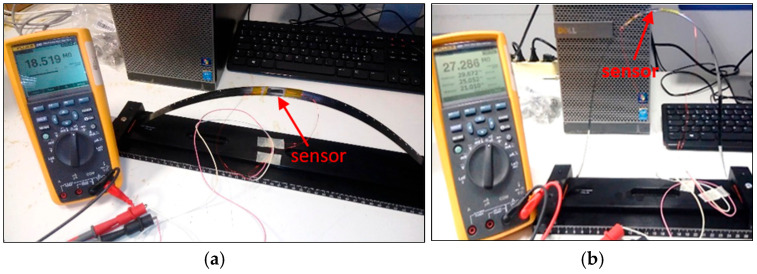
Sensor patch on steel beam: (**a**) resistance value of 18.5 MOhm at offset, (**b**) max curvature with 27.3 MOhm.

Another test is conducted by using a harmonic steel beam. The sensor has been bonded with the beam in a flat position. Once the beam has been installed in the sliding guide at an arbitrary initial curvature (Figure 12a), the resistance value changed to 18.5 MOhm (Figure 11b). This configuration has been considered to offset the strain at zero.

**Figure 13:**
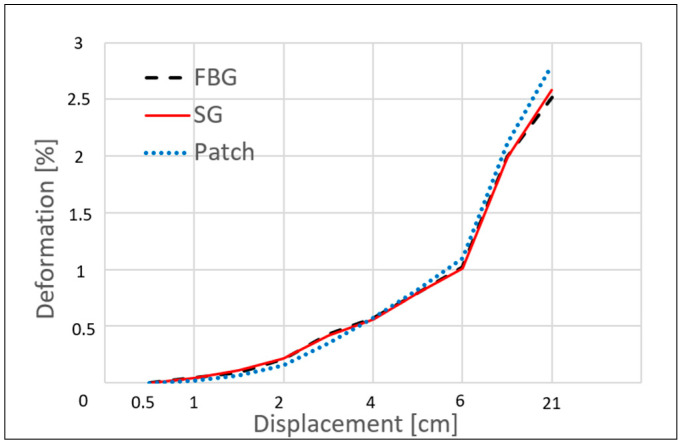
Strain values as a function of the edge distances.

The distance between the two edges is variable and measured by a millimetric guided slide (Figure 12a). This time, the experimental data are compared to a strain gauge and a fiber Bragg grating. The maximum curvature and strain are obtained close to the central position (Figure 12b). The values of displacement and the strain provided by the elastomer and the other two classical sensors are reported in Figure 13.

Two reference sensors (strain gauge and FBG) have detected consistent values with respect to the elastomeric patch within 1% of deformation. When curvature starts to increase at 21 cm, the elastomer seems to be able to withstand the more demanding curvature with a good linearity response. The bending tests highlighted the capability of the sensors to withstand low curvature radii, lower than 7.5 cm.

**Figure 14:**
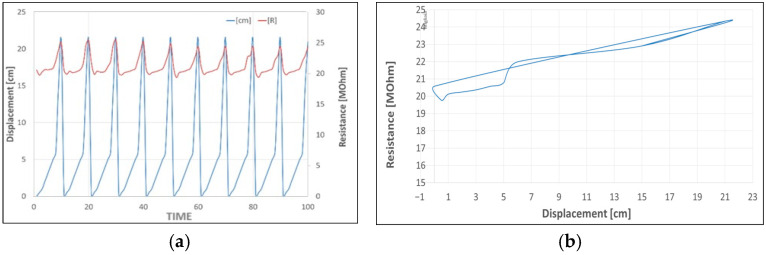
Displacement–resistance curves: (**a**) strain cycle values as a function of the edge position, (**b**) resistance variance as a function of the edge position.

A set of 10 cycles (Figure 14a) is repeated by sliding the beam edges in different positions from 0 to 21 cm, the distance. During the test, a resistance hysteresis is observed (Figure 14b).

**Figure 15:**
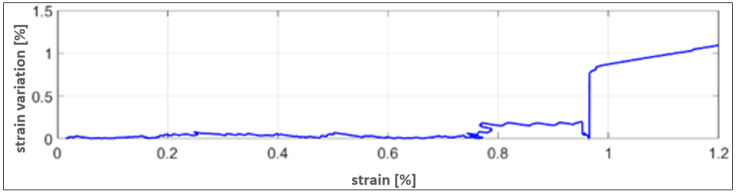
Delta strain patch values with respect to the SG reference.

This is evident in the Figure 15 reporting the difference between the strain measured by the elastomeric sensor and the strain gauge used as a reference. The delta strain highlights a growing deviation from the strain signal at 0.8%.

**Figure 16:**
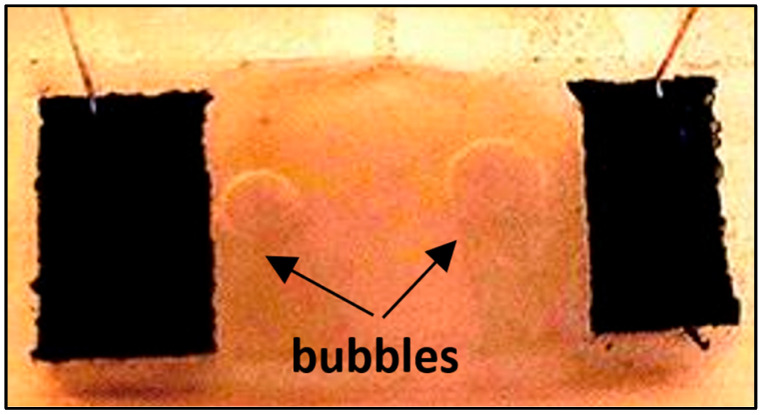
Visual inspection: evidence of bubbles at the interface.

Some bubbles at the elastomer interface have been observed during visual inspection. The bonding performance between adherend and adhesive depends on two main factors; including the surface roughness of the adherend and the bond line thickness of the adhesive. Such a defect may be associated with the progressive debonding and strain transfer losses. The debonding has been made more evident by using the post-processing contrast effect (Figure 16).

**Figure 17:**
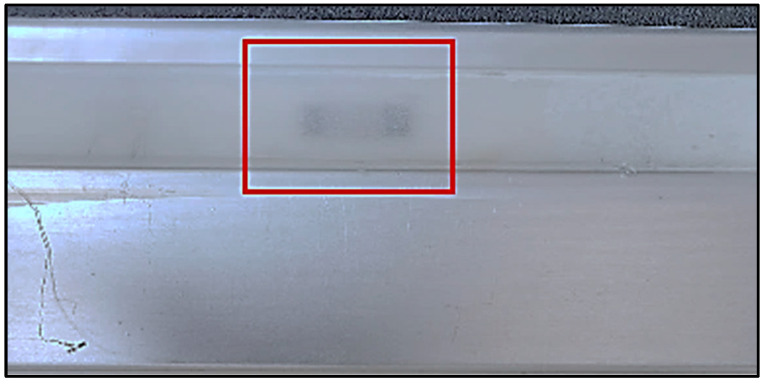
Morphing skin concept with aluminum, foam, and elastomer foil cover with one sensor spot (in the red frame).

The last test is realized with the target of observing the elastomer functionality in terms of resistive stability and structural adhesion under thermal conditions. The morphing structural element concept is made of metal segments and rubber elements (Figure 17).

**Figure 18:**
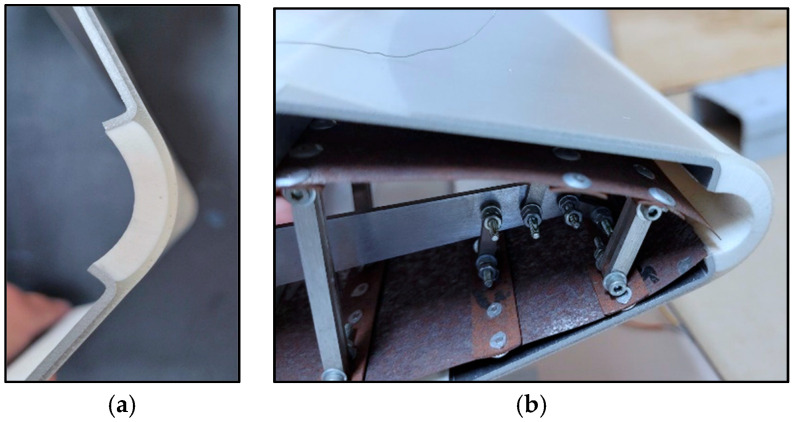
Morphing structural skin: (**a**) example of flexible skin for leading edge, (**b**) high curvature during chamber variation of a leading edge.

This skin element is protected by the elastomer film cover (Figure 18a,b) integrated by one graphene spot and provides a resistance value for a single purpose during temperature variation in the range from −20 °C up to +50 °C.

**Figure 19:**
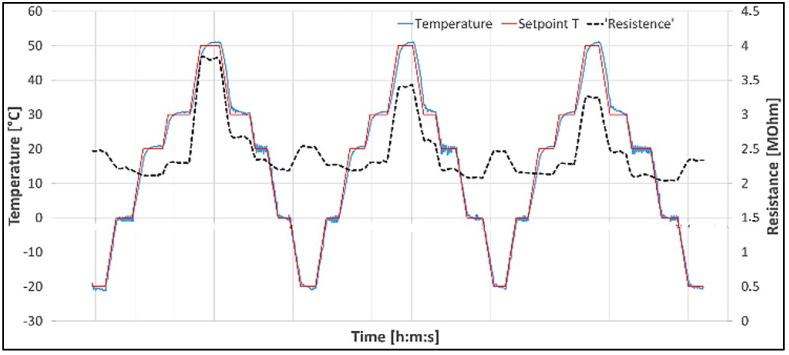
Morphing skin concept with elastomer sensor during thermal cycle.

The environmental chamber is provided by a temperature sensor (thermocouple K-type), used as a reference. The temperature set point has been defined by the control system to reach −20 °C and then reach +50 °C with intermediate steps at 0 °C, +20 °C, +30 °C, and +50 °C. A thermal load of 3 cycles is realized. Each cycle lasts 1 h and 20 min. Each plateau was 7 min long (Figure 19).

Appreciable variations of resistance are recorded between −20 °C and 50 °C. During the test, resistance hysteresis was probably due to a progressive assessment of the sensor microstructure, as also observed during the mechanical test. The most peculiar issue is the resistance value not following the set point; this is probably due to the different thermal inertia of the elastomeric matrix. More extensive tests must be conducted to better characterize the properties of the matrix. The resistance variance is reported in Table 1.

**Figure 20:**
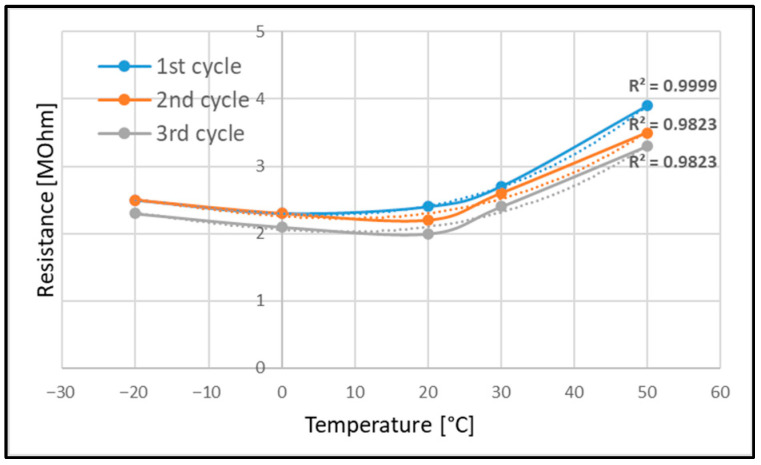
Resistance variance during the thermal cycles.

To better visualize the trend of resistance variation during the three cycles, a dedicated plot is provided in Figure 20. Having made it clear that the outcomes are related to only three cycles and hence cannot be a reference for stating a certain characterization, it is, however, nice to see a preliminary trend of the data.

It seems that the sensor material reaches a minimum value at 20 °C, and the R^2^ test shows an assessment of the trend from the second cycle on.

## 4. Conclusions

The paper reported preliminary outcomes from basic research activity (TRL < 3) specifically devoted to the realization of an elastomeric film cover to be bonded on an aerospace morphing airfoil characterized by a high curvature shape. Among the most critical applications in the morphing field, one recalls the possibility of integrating such a sensor at the leading-edge zone of a wing, characterized by extreme curvatures. The key innovations in design and fabrication include:

- Adoption of a custom-formulated, low-viscosity PDMS system crosslinked with TEOS and DBTDL, promoting improved graphene dispersion.
- Development of a novel sensor layout incorporating an insulating layer between the active material and the substrate.
- Integration of micro-scale voids at the interface to strengthen bonding with the substrate.
- A basic resistivity (zero strain) of 3.62 MOhm over a sensor 20 mm long (distance between the electrodes), 5 mm wide, and 1 mm thick.
- The sensor proved to withstand an imposed elongation of 30%.
- The bending tests highlighted the capability of the sensors to withstand low curvature radii, lower than 7.5 cm.
- Then, within the thermal characterization between −20 and +50 °C, a stability of the signal was observed.

All these features make the sensors a good candidate for laboratory prototypes of morphing concepts. Further improvements must be addressed not only to make the manufacturing process repeatable, but also to introduce multi-functionality like hydrophobic property. Moreover, such a film cover can be removed or substituted during maintenance for sensor repair.

## Figures and Tables

**Table 1:** Resistance delta variation as a function of the temperature for a flat and curved (loaded) panel.

Temp [°C]	First Cycle [MOhm]	Second Cycle [MOhm]	Third Cycle [MOhm]
−20	2.5	2.5	2.3
0	2.3	2.3	2.1
20	2.4	2.2	2
30	2.7	2.6	2.4
50	3.9	3.5	3.3

## Data Availability

The original contributions presented in this study are included in the article. Further inquiries can be directed to the corresponding author.
